# Probing the functional magnetic resonance imaging response to psilocybin in functional neurological disorder (PsiFUND): study protocol

**DOI:** 10.12688/wellcomeopenres.22543.1

**Published:** 2024-07-24

**Authors:** Matt Butler, Catherine Bird, Carolina Maggio, Amy Durden, Nadav Modlin, Kete Campbell-Coker, Mark Edwards, Susannah Pick, L.S. Merritt Millman, Emily Lowery, Chiranth Bhagavan, Richard Kanaan, Dawn Golder, Bridget Mildon, Mitul Mehta, James Rucker, Timothy R Nicholson

**Affiliations:** 1 Neuropsychiatry Research and Education Group, King's College London, London, England, UK; 2 Psychoactive Trials Group, King's College London, London, England, UK; 3 Department of Neuroimaging, King's College London, London, England, UK; 4 Psychological Sciences, King's College London, London, England, UK; 5 Department of Psychiatry, The University of Melbourne, Melbourne, Victoria, Australia; 6 Austin Health, Heidelberg, Victoria, Australia; 7 FND Hope, Salmon, Idaho, 83467, USA

**Keywords:** psychedelics, psilocybin, functional neurological disorder, fMRI

## Abstract

**Background:**

Functional neurological disorder (FND) is a common cause of neurological symptoms including paralysis, seizures, and movement disorders. It is often debilitating, is associated with high health and social care costs, and can have a poor prognosis. Functional magnetic resonance imaging (fMRI) has suggested FND is a multi-network disorder; the default mode network (DMN) may be specifically implicated. Converging evidence suggests that other variable mechanisms including dissociation, interoception, and motor agency may be differentially abnormal in people with FND. Psychedelics are currently under investigation for numerous neuropsychiatric disorders and have been shown to disrupt functional networks such as the DMN. Administering psychedelics to people with FND will help us to probe mechanistic theories of the disorder.

**Protocol:**

In this open-label neuroimaging study, we will administer 25mg oral psilocybin with psychological support to people with chronic FND (target n = 24). Participants will undergo resting-state and task-based (Libet’s clock, a measure of motor agency) fMRI sequences which will be compared in a pre-post manner. Additional mechanistic outcomes including measures of interoception (heartbeat tracking task), somatisation, illness perceptions, imaginative suggestibility, and dissociation will be collected. Data on expectancy, preparedness, and subjective experience of the psychedelic experience will also be gathered. Participants will be followed up for three months following psilocybin administration. fMRI changes in networks such as the DMN will be analysed using seed-based approaches, and additional exploratory analysis of resting-state imaging will take place.

**Discussion:**

The study will help us to probe the mechanisms thought to potentially underpin FND. As the first modern study of psychedelics in FND, it will also help us to understand whether psychedelic administration alongside psychological support might be safe and feasible in this patient population.

## Introduction

### Background

Functional neurological disorder (FND) produces a range of neurological symptoms and signs which have specific characteristics that positively separate them from those caused by neurological diseases and which implicate a specific malfunction of movement and perception ^1^.

FND is common ^2– 4^, with up to 100,000 in the UK living with the disorder ^5^. Disability is similar to other neurological disease ^6^. Symptoms are often found to persist or worsen over time ^4^, and prognosis can be poor ^7, 8^. Inpatient healthcare costs alone of FND in the United States are estimated to be around $1.2 billion per year ^9^. A large survey of people with FND indicated that only 15% were able to work full-time ^10^.

Current best-available treatment options are psychological therapies (such as cognitive behavioural therapy), psychoeducation, physiotherapy, and multidisciplinary rehabilitation, however these are not always effective ^4, 11, 12^. Although pharmacological therapies are not frequently used, there are specific cases in which psychoactive compounds may be effective as a single dose, particularly when used to augment psychological or physical therapy, for example in therapeutic sedation for functional dystonia ^13^ and historical cases of drug-facilitated abreaction ^14^.

### The predictive processing theory of FND

The increasingly dominant model of FND is that of a disorder of predictive processing ^15^. This model posits that the brain produces dynamic models of the body and the world through top-down expectations (known as ‘priors’) which are updated by bottom-up sensory input, to form ‘posteriors’. Thus, perception and experience are variable combinations of top-down expectations and bottom-up sensory information.

The predictive processing model has been used to conceptualise neuropsychiatric disorders. In chronic pain, a precise prior of pain is not updated by a non-nociceptive sensorium ^16^. In phantom limb syndrome, a precise prior of a painful limb is not updated by sensory information indicating amputation ^17^. In the case of functional leg weakness, for example, there may be an overly-precise prior of inability to move the limb, which is not updated from bottom-up sensory information indicating preserved anatomical and physiological function. Thus, the neurally-generated experience for the individual affected is an inability to move the limb.

### Mechanistic evidence in FND

Neurobiological theories of FND have been supported by neuroimaging ^18^. People with FND have shown increased connectivity in brain regions which comprise the default mode network (DMN), such as the medial prefrontal cortex and posterior cingulate cortex (PCC), both in resting-state and task based paradigms ^19– 25^. This increased DMN connectivity in patients with FND may be a correlate of symptom presence ^15^.

FND is also associated with impaired sense of agency. Agency refers both to the subjective feeling of volitional control one’s body, as well as the ability to enact such control ^26^. Research incorporating an agency paradigm (Libet’s clock test) has indicated that people with FND have a decreased awareness of the intention to move (motor agency) in comparison to healthy controls. In addition, replicated functional imaging studies in FND have found hypofunction of the right temporoparietal junction, a key node in the network proposed to underpin agency ^26– 30^.

Dysfunction of interoceptive processing may also contribute to the generation of functional symptoms ^31^. People with FND may have poorer interoceptive awareness than healthy controls, as shown via interoceptive tasks such as heartbeat tracking ^32– 36^. Difficulties with interoceptive processing may be a correlate of the disorder or may represent a means by which symptoms arise via aberrant interpretation of somatic signals.

Finally, dissociation may be another mechanisms by which symptoms occur ^33^. Dissociation can refer to the subjective feeling of disconnect from the self, others, or the environment (also known as depersonalisation and derealisation). It also has an additional specific definition as a psychological model which describes the splitting off of conscious awareness from attention ^37^. In FND, this may manifest with the feeling of loss of volitional control over a neurological function.

### Current psychedelic research

Classical psychedelics are agonists or partial agonists at the serotonin (5-HT) 2A receptors which induce states of acutely altered consciousness ^38^. Prior to the 1970s, psychedelics were successfully investigated for a number of neurological and psychiatric indications ^39– 41^. A resurgence of research interest in psychedelics in recent years has led to investigation in neuropsychiatric disorders such as treatment-resistant depression ^39^, headache disorders ^42^, anorexia ^43^, PTSD ^44, 45^, alcohol use disorder ^46^, tobacco addiction ^47^, existential distress in life-threatening disease ^48^, and obsessive-compulsive disorder ^49^. Psychedelics may have ‘cross-diagnostic potential’, and could target core elements of a shared psychopathology across neuropsychiatric disorders, including FND ^40^.

Contemporary psychedelic research has emerged within an environment of lingering cultural stigma as well as premature and unjustified hype about their medical efficacy ^50, 51^, however well-designed trials are attempting to address these issues ^52^. Safety data from these trials is reassuring ^41, 53^, and psychedelic drugs have been shown to be physiologically non-toxic and non-habit forming ^39, 54, 55^.

Modern psychedelic research involves non-directive psychological support ^39, 40, 56, 57^, in which the therapeutic relationship between participant and therapist is of central importance ^58^. This follows three phases: preparation, support during dosing and post-dose psychological integration ^57^. At doses of 25mg psilocybin, participants may have transient psychologically-challenging experiences ^59^, however psychological preparation can mitigate these ^60^, and post-dose integration helps to process the experience and build upon insights gained ^61^.

### Mechanistic effects of psychedelics

5-HT _2A_ receptors are densely distributed in neural networks such as the DMN ^62^ and psychedelics have been shown in fMRI studies to reduce the functional connectivity of neural networks such as the DMN. The extent of reduction in connectivity correlates with self-reported degree of loss of selfhood (‘ego-dissolution’) ^63^. Functional neuroimaging in psychedelics have shown relatively consistent effects of decreases in within-network-connectivity and increases in between-network-connectivity in canonical brain networks including the DMN ^64^. 

Acute administration of psychedelics may temporarily flatten the brain's predictive processing hierarchy, providing a window during which aberrant priors can be recalibrated ^65^. Loosened global network connectivity may enable improvement of neuropsychiatric symptoms by reducing the influence of top-down processing and loosening established prediction errors ^66^.

Psychedelics have been shown to exert significant changes on the acute sense of body ownership ^67, 68^. Although direct research on psychedelic and agency is scarce, psychedelic users have been shown to have agency related measures which are no different to healthy controls ^68^. Similarly direct research analysing the effects of classical psychedelics on interoception is lacking, however the psychoactive compound salvinorin-A has been shown to modify interoception ^69^. Randomised trials of 3,4-Methylenedioxymethamphetamine (MDMA)-assisted psychotherapy for treatment of chronic posttraumatic stress disorder showed non-significant reductions in dissociative experiences in the treatment arms ^70^.

### Psychedelics in FND

Previous authors have suggested FND as a target disorder for psychedelics ^41, 71^, and systematic reviews of ‘pre-prohibition’ case series of patients with FND administered psychedelics such as psilocybin and lysergic acid diethylamide (LSD) have found that side effects were minimal and transient in this population ^72, 73^. Nevertheless, as all relevant studies were conducted with insufficient experimental designs, further modern feasibility work is required.

In an international survey of people with FND, self-reported physical side-effects from recreational psychedelic use (n=10) were minimal at worst, and 46% of the >1,000 respondents reported that they would be willing to take part in medically-supervised psychedelic studies, with a further 20% undecided ^74^.

### The proposed study

Taken together, this evidence suggests that psychedelics may target some of the mechanistic processes which underpin FND. Administering the psychedelic psilocybin to people with FND will allow us to probe theories of FND which postulate that it is a disorder typified by altered network connectivity across brain networks, including the DMN. Using a pre-post design, we will be able to test whether impaired sense of agency, changes in interoception, and dissociative phenomena are modifiable features of the disorder. 

## Protocol

### Aim

The purpose of this study is to investigate the functional neuroimaging response to a single dose of psilocybin in patients with functional neurological disorder.

The study is pre-registered on clinicaltrials.gov (NCT05723276). The study and all relevant documents have ethical approval from the Health Research Authority Research Ethics Committee (23/WA/0213).

### Objectives

We will administer a single 25mg oral dose of psilocybin in people with functional neurological disorder. Participants will undergo functional resting-state and task-based (Libet’s Clock Test) fMRI one day prior to and one week following the psilocybin administration.

Exploratory objectives include assessment of the effect of psilocybin administration on mechanistic targets including interoceptive ability, dissociative phenomena, illness perceptions and self-compassion.

### Endpoints

***Primary endpoints***

The primary endpoint of this study will be change in functional connectivity functional resting-state imaging via within-subjects region of interest connectivity analysis using a priori seed regions (medial prefrontal cortex, subgenual anterior cingulate cortex, posterior cingulate cortex, temporo-parietal junction) and task-based magnetic resonance using Libet’s clock test. Additional exploratory functional neuroimaging analysis will likely take place with other methodologies (e.g. measures of modularity, entropy).

***Exploratory endpoints***

1. To assess any changes in the following outcomes following psilocybin administration (pre-post design):
  a. Interoceptive accuracy and confidence (as per the Heartbeat tracking task and the Multidimensional Assessment of Interoceptive Awareness – Version 2)
  b. Dissociation (Multiscale Dissociative Inventory Scale)
  c. Imaginative suggestibility (Creative Imagination Scale)
  d. Illness perceptions (Brief Illness Perceptions Questionnaire)
  e. Somatisation tendencies (Somatoform Dissociation Questionnaire)
  To explore the association of primary and exploratory outcomes with other measures, including:
*i.*  Expectancy (as per the Stanford Expectations of Treatment Scale)
*ii.*  Psychedelic preparedness (as per the Psychedelic Preparedness Scale)
*iii.*  Acute psychedelic experience intensity (as per the Ego Dissolution Inventory and the 5-Dimensional Altered States of Consciousness Rating Scale)
*iv.*  Self-compassion (as per the Self-Compassion Scale)
*v.*  Personality structure (as per the Short Assessment of Personality Scale)
2.  To explore correlations of functional MRI connectivity with clinical outcomes (via the Clinical Global Impression; CGI) and assessment of associated symptoms (via the Revised Fibromyalgia Impact Questionnaire; FIQR).
3.  To explore participants views on acceptability on psychedelic administration with psychological support via qualitative interviews.

## Hypotheses under investigation

**Figure 1.**
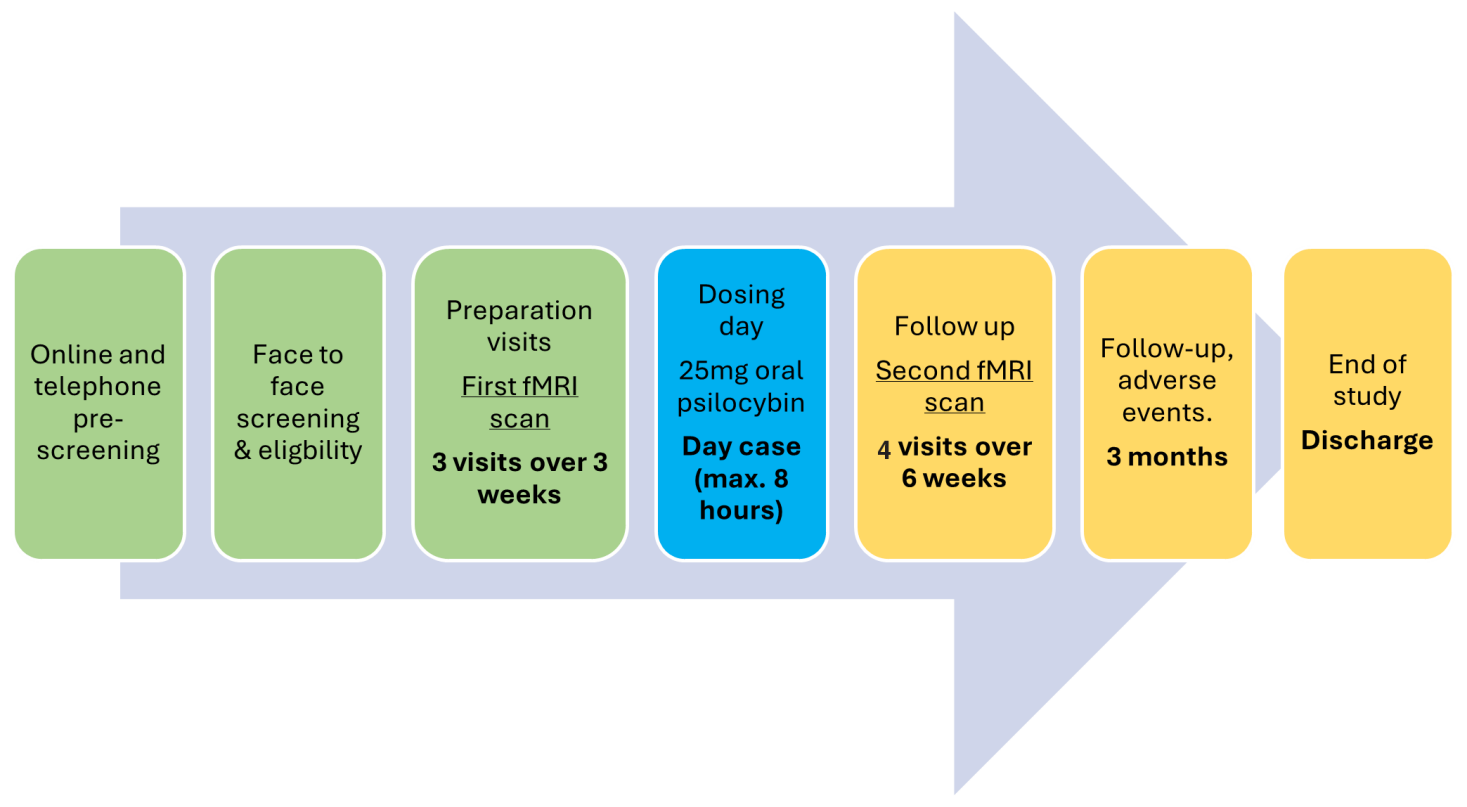
Study flowchart.

- Hypothesis 1: Networks implicated in FND such as the default mode network (DMN) and limbic-motor cortex connectivity will be altered post psilocybin. The direction of change will be towards that of healthy controls ( Figure 1).
- Hypothesis 2a: Changes in in other mechanistic markers, including motor intentionality, interoception, dissociation, imaginative suggestibility, and somatisation will be observed in the direction of normative controls.
- Hypothesis 3: There will be improvement in the FND core and associated symptom severity as measured by the CGI and FIQR.
- Hypothesis 4: Qualitative interviews will suggest acceptability for this form of therapy. Themes which emerge from these interviews will include changing relationships to their disorder, increased self-compassion, and meaningful experiences engendered by trial participation.

### Study design

This study will be an open label within-subjects design in up to 24 participants who have a diagnosis of FND as per Diagnostic and Statistical Manual of Mental Disorders (5 ^th^ Edition) (DSM-5) criteria ^75^. They must have failed to respond to an FND-specific treatment (such as CBT, physiotherapy, multidisciplinary rehabilitation). Within the confines of the sample size, a representative sample of motor and seizure subtypes will be sought for inclusion.

### Sample size calculation

We will recruit up to 24 participants for this study. The primary aim of the study is to assess resting-state and task-based functional neuroimaging changes. Based on a previous fMRI study of the effect of psilocybin on resting-state connectivity obtained by fMRI, a mean response in connectivity in the default mode network (a connectivity network of interest) of 2.48 (arbitrary units) and a standard deviation of 2.60 ^76^ was found in pre versus post psilocybin within-subjects analysis; this signifies an anticipated minimum detectable effect size of 0.95. For a two-tailed test and an alpha of 0.01 (to account for multiple testing) and with a power of 0.90, a total sample size of n=20 is required. An additional four participants will be recruited given expected dropouts.

### Recruitment

Recruitment will be via a number of methods, including referral from secondary and tertiary care services as well as established registries of patients who have consented to contact from study teams, word of mouth, and directly from advertising in the community and on social media. The public-facing study website (psifund.co.uk) will contain information about the study (including the participant information sheet), contact details for the study team, and frequently asked questions. All patient-facing information has been reviewed and approved by the Research Ethics Committee.

### Screening

Potential study participants will be initially screened by online pre-screening survey. The online pre-screening will prompt potential participants to read the Participant Information Sheet and indicate their consent for providing information on their diagnosis, demographics and contact details. Screening questions will include the inclusion and exclusion criteria in lay terms, which respondents will need to indicate with a checkbox that they satisfy. We will also ask for a telephone contact number and an email address. Phone screening will then be undertaken to clarify inclusion and exclusion criteria and to arrange face to face screening if appropriate. Additionally, participants may be directly referred via contributing Participant Identification Centres.

Those who are deemed to be potentially eligible will be invited for a face-to-face screening assessment with a member of the study team. Final study inclusion and consent will be taken by a study medic.

The following inclusion criteria will apply:

1) Age 25 – 60 years.
2) Fluent in the English language
3) A diagnosis of FND from a neurologist and/or neuropsychiatrist as per DSM-5 criteria
4) Moderate or severe symptoms (≥4 on Clinical Global Impression Severity (CGI-S) scale) which have been present for >12 months and have failed to respond to a recognised treatment.
5) Able to tolerate fMRI scanning procedures.

**Failed to respond** is defined as an inadequate (i.e. not complete) response to a full course of FND-specific therapy, such as CBT, physiotherapy, or inpatient treatment. Therapy must have been undertaken by a suitably trained expert in FND and must have been specifically targeted at FND symptoms.

The following exclusion criteria will apply:

1) Diagnosis of severe depression. *
2) Diagnosis of bipolar affective disorder. *
3) Diagnosis of a psychotic disorder, EXCEPT substance/medication induced psychotic disorder where the duration was limited to the acute period of direct intoxication with the substance/medication. *
4) Diagnosis of drug or alcohol dependence disorder. *
5) Diagnosis of a personality disorder. *
6) Diagnosis of any dementia based on clinical interview by a psychiatrist.
7) Diagnosis of any autistic spectrum disorder based on clinical interview by a psychiatrist.
8) Diagnosis of any learning disability based on clinical interview by a psychiatrist.
9) Significant suicidal behaviour in past 12-months defined using the Columbia-Suicide Severity Rating Scale (C-SSRS). *
10) Any other factor which would render the participant unsuitable for psilocybin and/or interfere with a supportive therapeutic relationship and/or preclude safe follow-up.
11) Those unable to give informed consent.
12) Medical diagnosis incompatible with psilocybin treatment (see *below*).
13) Inability to provide a screening blood sample, urine sample, or electrocardiogram.
14) Biochemical abnormalities (defined as falling outside the normal reference range) as evaluated by a full blood count, full biochemistry profile and thyroid function tests. Biochemical abnormalities must also be determined as clinically significant by a medical doctor to fulfil the criterion for exclusion.
15) Electrocardiographic abnormalities determined as clinically significant by a medical doctor.
16) Women of childbearing potential not using contraception.
17) Pregnant or breast-feeding women.
18) Non-registration with a GP or failure to consent to sharing of the GP summary care record and any psychiatric assessments held.
19) Those enrolled in another clinical or research study.
20) Use of any psychedelic substances >2 times in past 12 months.
21) Any factor which would exclude the participant from MRI scanning (e.g., presence of metal).

Diagnoses will be defined in each case as meeting DSM-5 criteria, where relevant.

*In all highlighted cases, diagnoses will be screened via appropriate tools (such as the Mini Neuropsychiatric Inventory v7.0 [MINI] ^77^) and will be subject to confirmation at clinical interview by a psychiatrist.

To be eligible for this study participants will need, in addition to satisfying the eligibility criteria, to agree to the following:

1) Provide contact details of a trusted friend or relative that the study team may contact in the event of an emergency.
2) To stay within the boundaries of the research centre during the Dosing Visit for at least six hours after the psilocybin is given, or until it is clinically determined that they are safe to leave, whichever is longer.

### Exclusions for pre-existing medical conditions

Participants will be excluded if they have a current diagnosis of ≥1 of:

- Uncontrolled diabetes
- Hypertension (defined as a systolic blood pressure ≥ 160mm/Hg or a diastolic blood pressure ≥100mm/Hg on three separate readings). All readings of systolic blood pressure ≥ 140mm/Hg or diastolic blood pressure ≥ 90mm/Hg will be reviewed by a clinician. Hypertension ascertained prior to dosing will be subject to clinical confirmation via collateral information from the GP or other source.
- Cardiac failure, defined as class IV of the New York Heart Association classification
- Renal failure, defined as ≥ stage 4 (GFR ≤ 29mL/min)
- Liver failure, defined as a clinical diagnosis of liver fibrosis, cirrhosis of the liver, liver failure or advanced liver disease.
- Any cardiac arrhythmia, except atrial fibrillation.
- Any form of epilepsy

Past diagnosis of ≥1 of:

- Cerebrovascular accident or intracerebral trauma.
- Myocardial infarction within 1 year prior to the screening visit.

***Women of childbearing potential***

A female who is not of childbearing potential is considered to be postmenopausal (at least 12 consecutive months without menstruation) or permanently sterilized (e.g., hysterectomy and/or bilateral salpingectomy).

For females of childbearing potential who may participate in the study, the following methods of contraception, if used properly and used for the duration of the study, are considered sufficient:

- Oral contraceptives
- Patch contraceptives
- Injection contraceptives
- Diaphragm or cervical cap with spermicide
- Vaginal contraceptive ring
- Intrauterine device
- Surgical sterilization (hysterectomy and/or bilateral salpingectomy)
- Vasectomized partner
- Sexual abstinence
- Same-sex partners

Periodic abstinence, i.e., calendar, symptothermal, or post-ovulation methods, and tubal ligation/occlusion are not an acceptable form of contraception for this study.

The investigator and each participant will determine the appropriate method of contraception for the participant during the participation in the study. This will be documented at Screening (V1a).

### Study phases

**Figure 2.**
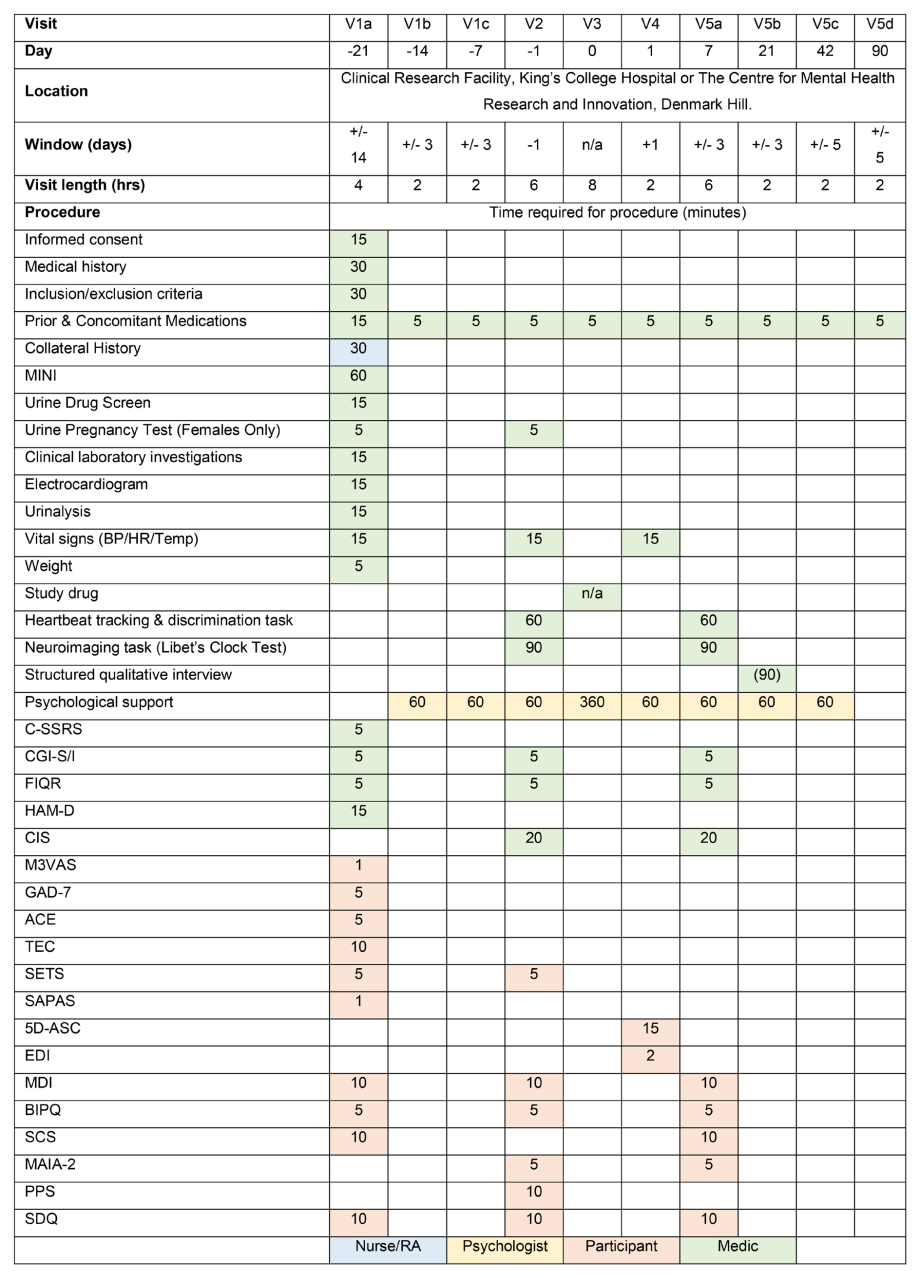
Schedule of events. Estimated time needed for completion of each task indicated in the table in minutes.

Please refer to the study flowchart ( Figure 1) and breakdown of study visits ( Figure 2).

***Screening and preparation phase***

Participants will be assessed by a study psychiatrist and enter a Screening & Preparation phase of between one to eight weeks. All participants will receive the same screening assessments and baseline psychological preparation session prior to receiving the dose.

Withdrawal from relevant medications will be supervised by a clinician, flexible to the needs of the participant. This step is required because many medications, particularly those with serotonergic effects, may attenuate or block the effects of psilocybin. Participants will be allowed to continue to take medications for other medical illnesses throughout the study and on the Dosing Visit, provided they do not interact unfavourably with psilocybin.

We will allow the prescription of hypnotic medication (zopiclone, zolpidem or zaleplon within BNF dosage) for insomnia or a benzodiazepine (lorazepam or diazepam) or antihistamine (promethazine) for anxiety or withdrawal phenomena. Other over-the-counter remedies will be allowed, except for St John’s Wort. Of note, a recent systematic review found no evidence to suggest significant interactions between psilocybin and psychiatric medications, including antidepressants ^78^.

During the Screening & Preparation Phase, participants will be reviewed weekly, either face to face or online. We will extend the screening and preparation phase for a further period of up to eight weeks if participants require more time to withdraw from their medication, or if collateral history suggests a medical or personal problem that has not been adequately investigated, treated, or accounted for, or if participants require more preparation with their therapists prior to the dose.

During the screening phase, participants will be introduced to a therapist and a chaperone familiar with psilocybin and its effects. The therapist and/or chaperone will provide psychoeducation, establish rapport, and address anxieties prior to the dose; to provide appropriate care and support to participants during the dose; to provide an opportunity for psychological support and integration after the experience. Depending on participant needs we will arrange additional sessions for psychological preparation and integration as required.

***Baseline, dosing, and day one visit***

One day prior to the Dosing Visit, participants will be invited to attend for the Baseline Visit. Final eligibility will be confirmed at this visit. We will perform baseline clinical procedures and measures of outcome and functioning ( Figure 2). Participants will meet with their therapist and chaperone to prepare them for the Dosing Visit.

At this visit, participants will undergo the first fMRI scan. This scan will include structural, resting-state, and task-based (Libet’s Clock) sequences. Outside of the scanner, participants will also undertake a heartbeat tracking test, a measure of interoceptive accuracy & awareness.

The Dosing Visit will take place 1–3 days after the Baseline Visit. Participants will receive 25mg of oral psilocybin. Neither participant nor study team will be blinded (i.e. the study is open label). The study team will perform a brief mental state exam, before allowing sufficient time for participants to relax and for any anticipatory anxieties or practical considerations to be addressed.

The environment within which the dose will be given will be a quiet, neutrally furnished room with adjustable lighting. Participants will lie or sit down on a bed, and easy access to the lavatory and light refreshments will be available. Participants will be given an eye mask and earphones through which they will be able to hear a curated playlist of relaxing music. The therapist and chaperone will be present in the room, with at least one person with the participant at all times during the Dosing Visit.

After the administration of the study drug, we will require participants to stay within the boundaries of the research facility until it is clinically determined that they are safe to leave. The subjective effects of psilocybin peak at approximately two hours and then wear off over approximately 2–4 hours. Inter-individual variation is, however, expected and participants will be accompanied for as long as necessary by the study team. If necessary, overnight hospital admission will be arranged for observation, with review by the study team the following morning and reporting of the event to appropriate pharmacovigilance authorities.

If necessary, and as a last resort, in the event of ongoing extreme distress unresponsive to therapist intervention, or in the event of threatened or actual violence unresponsive to de-escalation techniques, rescue medication will be given in liaison with the study medical and nursing staff. We will use oromucosal midazolam 2.5mg-5mg orally as a first line. Any further treatment will follow NICE guidelines. This will be given under the supervision of a psychiatrist.

Participants will attend the research facility the day following dosing. Participants will complete self-reported measures. Participants may meet with their therapist for up to 90 minutes to discuss their experience, and for example to process any difficult material that may have arisen.

***Follow-up phase***

The initial follow-up phase will last for six weeks post-dosing. During this time, the participant will attend three face-to-face follow-up visits which will include psychological integration sessions. The final three-month follow-up visit may take place online (via Microsoft Teams) if deemed appropriate by the participant and study team.

At one week post dose, participants will undergo follow-up fMRI imaging. The scanning protocol is the same as the first fMRI scan. An optional semi-structured qualitative interview about participants’ experiences and opinions on the study and therapy will also take place during follow-up. We will ask participants to complete self-report measures and a member of the study team will perform study team-rated measures ( Figure 2).

### Psychological support

The psychological support used in this study will be based on a model that has been developed separately for COMPASS Pathways sponsored trials in depression. The psychological model is divided into three phases: preparation, dosing, and integration. This therapy is not specific to FND but will allow for the safe administration of psilocybin using a framework which has been successful in trials of psychedelics in other neuropsychiatric disorders. The therapists in this study have been trained as part of existing psilocybin studies and have experience of working with medical psychedelics.

Preparation focusses on building mutual trust, active listening, psychoeducation about the drug experience, and teaching methods of coping with difficult or unusual experiences that might arise. We will require participants to undergo a minimum of three hours of preparation, which will be delivered over several separate sessions. Satisfactory completion of this preparation will be assessed prior to dosing and the amount of preparation recorded in the case record form.

### Neuroimaging task

Neuroimaging will take place on a Premier 3T MRI scanner. Scans will be clinically reviewed by a reporting clinician as routine. Participants will have a basic T1-weighted structural scan, followed by a resting-state fMRI, during which time they will be asked to lie back quietly with their eyes shut. Following this, participants will have functional MRI whilst they undertake Libet’s clock test.

In Libet’s Clock Test, participants will watch a ball revolving around a clock face and will press a button after a random time interval and will be asked (in 2x2 blocks of 15 trials) to note when they felt the urge to press (W judgement) or when they actually pressed the button (M judgement). The ball then appears at a random position, and participants are required to return it to the position it was in when they felt the urge to press the button, or actually pressed it depending on the block. Average latencies of the W and M judgements will be calculated as the differences between the final position of the ball and its position at the time of the recorded button press ^79^.

### Interoception task

We will use a modified heart-beat tracking task to measure interoceptive accuracy and awareness. This is a version of the heartbeat tracking task developed by Schandry (1981) ^80^ and modified with previous successful use by Pick *et al.* (2020) ^33^ and Millman *et al.* (2023) ^36^. Participants are advised not to wear any devices which would facilitate heartbeat tracking, and are asked not to explicitly measure their pule (e.g. via radial pressure).

Participants will be asked to sit comfortably and attend to and silently count their own heartbeats during four intervals of 25, 30, 35 and 40 seconds or 29, 34, 39 and 44 seconds.. The tracking trials will be interspersed with 20s rests in which participants fixated on the computer screen. The start and end of each heartbeat tracking, and rest period will be indicated on screen ‘Start counting your heartbeats’ in green, and ‘Stop counting’ in red. Immediately after each tracking period, participants will be cued to type in the number of heartbeats they had counted, followed by a confidence rating (0–10, low-high certainty). Participants will be advised to indicate 0 if they could not feel any heartbeats at all.

Participants will be attached to a 2-lead ECG throughout the task to record actual heart beats, which will be recorded using a data acquisition system (PowerLab). Participants will be explicitly asked not to attempt to monitor the passing of time by counting seconds. A practice trial will be completed by all participants before starting the experimental procedures. Interoceptive accuracy will subsequently be calculated using the following formula:

     1/4 ∑ [(1 − (|actual heart-beats – perceived heart-beats | / actual heart-beats)

Following the heartbeat tracking task, participants will complete the time estimation task. This requires participants to count seconds during four randomised intervals (27, 32, 37 and 42 seconds, or 23, 28, 33 and 38 seconds). Otherwise, the trial will be conducted in the same manner as the heartbeat tracking task above. Time estimation accuracy will subsequently be calculated using the following formula:

     1/4 ∑ [(1 − (|actual seconds – perceived seconds | / actual seconds)

***Multidimensional Assessment of Interoceptive Awareness – Version 2 (MAIA-2)***

In parallel, we will collect the Multidimensional Assessment of Interoceptive Awareness – Version 2 (MAIA-2) which is an 8-subscale trait self-report questionnaire to measure multiple dimensions of interoception (awareness of bodily sensations). The MAIA-2 is suitable for adults and has 37 items ^81^.

### Semi-structured qualitative interview

Participants will be offered an optional semi-structured interview at a visit following the dosing. The interview will be conducted by a clinician in the trial team and will principally focus on the experience of the psilocybin dosing session as well as on any subsequent positive or negative effects on FND symptoms. The first stage of the interview will focus on illness background and perceptions, whilst the second stage will focus on participant’s own appraisal of the effects (if any) of the experience of the dosing session.

### Clinical reported outcome measures

***The Columbia Suicide Severity Rating Scale (C-SSRS)***

The C-SSRS is a clinician- and participant-rated scale that includes definitions of suicidal behaviour adapted from the Columbia Suicide History Form ^82^. The purpose of this scale is to exclude participants with significant suicidal thoughts or behaviour where necessary. The CSSRS will be administered at screening.

***17 Item Hamilton depression rating scale (HAM-D-17)***

The HAM-D-17 is a 17-item scale used to measure the degree of symptom severity in depressed patients ^83^. The HAM-D-17 will be administered at screening and follow-up. The purpose of including this scale is to exclude severe depression in potential participants.

***Clinical Global Impressions Scale (CGI)***

The CGI severity and improvement scales offer a readily understood, practical measurement tool that can be administered by a clinician in clinical trial settings. The Scale has two sections: Severity of illness, and (relative) Improvement ^84^. We will undertake the CGI as part of the screening process as well as at follow-up visits.

***Revised Fibromyalgia Impact Questionnaire (FIQR)***

The FIQR is an instrument used in the evaluation of fibromyalgia patients. It assesses three domains: function, overall impact, and symptoms ^85^. There is significant overlap between the domains assessed by the FIQR and the ‘associated’ symptoms of FND, which are found at high rates and are found to be just as functionally impairing as the primary FND symptoms ^10^. We will undertake the CGI as part of the screening process as well as at follow-up.

***Creative Imagination Scale (CIS)***

The Creative Imagination Scale is a measure of imaginative suggestibility. It does not require a hypnotic/trance induction procedure ^86^.

### Participant reported outcome measures

***Stanford Expectations of Treatment Scale (SETS)***

The SETS is used in clinical trials to improve statistical sensitivity for detecting responses to interventions. The six-item SETS contains two subscales: positive expectancy and negative expectancy. The SETS is simple to administer, score, and interpret ^87^. We will use the SETS score to investigate whether expectancy is associated with any outcome changes following psilocybin.

***Psychedelic Preparedness Scale (PPS)***

The PPS is a 20-item self-report measure of the degree to which respondents feel prepared for a psychedelic experience. It measures four domains: Knowledge-Expectation, Psychophysical-Readiness, Intention-Preparation, and Support-Planning. Participants rate their agreement with statements on a seven-point scale from ‘not at all’ to ‘completely’ ^88^.

***5-Dimensional Altered States of Consciousness Rating Scale (5D-ASC)***

The 5D-ASC is a 94-item self-report measure of the acute effects of psychoactive drugs using 5 primary dimensions. Participants are given a statement pertaining to an element of conscious experience then asked to mark a point on a line between ‘No, not more than usually’ and ‘Yes, much more than usually’. The 5 dimensions are ‘oceanic boundlessness’, ‘anxious ego dissolution’, ‘visionary restructuralisation’, ‘auditory alterations’, and ‘reduction of vigilance’ ^89, 90^. We will use the 5D-ASC score to investigate whether subjective intensity of experience is a associated with any outcome changes following psilocybin.

***Ego Dissolution Inventory (EDI)***

The EDI is a short 8-question visual analogue scale aimed to assess the extent to which participants felt they had lost their sense of self (‘ego dissolution’) during an experience. The scale has been validated and demonstrates a close relationship between ego-dissolution and the psychedelic experience; it was explicitly designed to facilitate the study of the neuronal correlates of ego-dissolution ^91^.

***Multiscale Dissociation Inventory (MDI)***

The MDI is a 30-item self-report test of dissociative symptomatology. It is fully standardized and normed, and measures six different type of dissociative response ^92^. We will use the MDI score to investigate whether measures of dissociation change following psilocybin administration.

***The 20-item Somatoform Dissociation Questionnaire (SDQ)***

The 20-item Somatoform Dissociation Questionnaire evaluates the severity of somatoform dissociation ^93^. We will investigate whether any alterations in neuroimaging measures (fMRI functional connectivity) are linked to changes in dissociation measures following psilocybin.

***Brief Illness Perceptions Questionnaire (BIPQ)***

The Brief Illness Perception Questionnaire (Brief IPQ) is a 9-item questionnaire designed to rapidly assess cognitive and emotional representations of illness ^94^. The Brief IPQ uses a single-item scale approach to assess perception on a 0–10 response scale. It is developed by forming one question that best summarises the items contained in each subscale of the Illness Perception Questionnaire-Revised which has over 80 items. The Brief IBQ comprises five items on cognitive representation of illness perception: consequences, timeline, personal control, treatment control, and identity.

***Self-Compassion Scale (SCS)***

The Self Compassion Scale is 26-item scale comprising 6 subsections that tend to load together. Self-compassion entails being kind and understanding toward oneself in instances of pain or failure rather than being harshly self-critical; perceiving one’s experiences as part of the larger human experience rather than seeing them as isolating; and holding painful thoughts and feelings in mindful awareness rather than overidentifying with them ^95^.

***Maudsley 3-Item Visual Analogue Scale (M3VAS)***

The Maudsley Visual Analogue scales indicate the quality of mood, experience of pleasure and experience of suicidal thoughts or feelings during a preceding interval of time that can be varied according to study need ^96^. The MVAS will be administered at screening.

***Generalised Anxiety Disorder Scale (GAD-7)***

The GAD-7 is a self-rated screening and symptom severity measure covering 7 of the most commonly occurring anxiety symptoms ^97^. Participants choose one of four severity scores and also indicate the degree to which these problems caused functional and/or social difficulties. Scores are derived from the values for each of the 7 domains. The GAD-7 will be collected at screening.

***Adverse Childhood Experiences (ACE)***

The ACE is a 10 item questionnaire that collects data about childhood traumatic life events ^98^. The CTQ will be collected at screening.

***Traumatic Experiences Checklist (TEC)***

The Traumatic Experiences Checklist is a self-report measure addressing potentially traumatising events. It is a reliable and valid self-report instrument that can be used in research. Scores can be calculated for emotional neglect, emotional abuse, physical abuse, sexual harassment, sexual abuse, and bodily threat from a person ^99^. The TEC will be collected at screening.

***Short Assessment of Personality Scale (SAPAS)***

The SAPAS is a very short assessment of personality designed to capture indicative elements of personality that may further indicate personality structures captured within the ICD-10 and DSM-IV constructs of personality disorder. Eight items are rated ‘yes’ or ‘no’ ^100^. We will collect the SAPAS at screening to assess whether answers to any particular question are indicative of positive or negative response to psilocybin. The SAPAS will be collected at screening.

### Adverse events reporting

Data on adverse events will be collected and reported via the Health Research Authority safety reporting for non-CTIMP studies guidance ^101^. We require consent from any participants to additionally share anonymised adverse event information with COMPASS Pathways, who will provide the study drug.

### Analysis

***Functional neuroimaging***

Analysis of the neuroimaging data will take place using the KCL Neuroimaging Network and the Rosalind cluster. fMRI analysis will be carried out within Statistical Parametric Mapping software using a seed-based approach implemented in the CONN-fMRI Functional Connectivity toolbox. Resting state region of interest connectivity analysis will be performed using a priori seed regions (medial prefrontal cortex, subgenual anterior cingulate cortex, posterior cingulate cortex, temporo-parietal junction). Additional exploratory functional neuroimaging analysis will likely take place with other methodologies (e.g. measures of modularity, entropy).

***Study and intervention feasibility parameters***

Feasibility parameters will be assessed using descriptive statistics and confidence intervals. We will assess the rate of consent of eligible patients, study retention, loss to follow-up, the acceptability of psilocybin and deviations from study protocol. Confidence intervals will be estimated where appropriate.

Participant demographic and clinical characteristics will be summarised at baseline. Patterns of missing data or nonattendance will be described for the outcome at measurement time- points and reasons for drop-out. Statistics derived will be means and standard deviations or medians, minimum, maximum, and interquartile range for continuous measures.

### End of study

The study is expected to be open for recruitment for two years. The total duration of a participant’s involvement in the study will vary between 15 and 22 weeks, dependent on the length of the (clinically flexible) Screening & Preparation Phase. The follow up of participants will last for 3 months after the Dosing Visit. Ongoing care for participants will be handed over to primary or secondary care teams after this time.

## Discussion

This study will likely be the first in the modern era to administer psychedelics to people with FND. The primary aim is to assess functional neuroimaging changes following psychedelic administration. This may help us to further understand the complex pathophysiology underpinning FND, which is currently poorly understood ^4^. Additional putative mechanistic aspects of FND, such as dissociation, interoception, and agency will also be explored, and administration of psilocybin will allow us to probe whether these are modifiable targets.

Drug-facilitated therapy is current under review for a number of neuropsychiatric disorders, and there has been some emerging evidence that people with FND may respond to (es)ketamine therapy, which sometimes includes a psychotherapeutic component ^102– 104^. Response may be based on the ability of ketamine to disrupt brain networks or modify the top-down priors implicated in the disorder.

Pharmacological therapies are generally not specifically recommended for FND, however drug facilitated psychological ^14^ and physiotherapeutic ^13^ interventions do have a small evidence base in the disorder. The present study is not equipped to answer clinical questions about the efficacy of psychedelics; however, it may offer preliminary information about whether drug-facilitated psychotherapy is safe and feasible in this patient population. 

## Data Availability

No data are associated with this article.
