# Supplementary material for: Probing the functional magnetic resonance imaging response to psilocybin in functional neurological disorder (PsiFUND): study protocol
Source: Wellcome Open Res. 2025 Apr 22;9:401. Originally published 2024 Jul 24. [Version 2] doi: 10.12688/wellcomeopenres.22543.2 (PMC11450546; doi:10.12688/wellcomeopenres.22543.2)
Supplement: Peer review history

# Reviewer report: Reviewer response for version 2

**DOI:** 10.21956/wellcomeopenres.26488.r122474

**Published:** 2025-04-24

**Authors:** Manoj Doss

**Affiliations:** 1 Department of Psychiatry and Behavioral Sciences, The University of Texas at Austin Dell Medical School, Austin, USA

The authors have addressed my concerns, and I approve it now.

Is the study design appropriate for the research question?

Partly

Is the rationale for, and objectives of, the study clearly described?

Partly

Are sufficient details of the methods provided to allow replication by others?

Partly

Are the datasets clearly presented in a useable and accessible format?

Not applicable

Reviewer Expertise:

NA

I confirm that I have read this submission and believe that I have an appropriate level of expertise to confirm that it is of an acceptable scientific standard.

# Reviewer report: Reviewer response for version 1

**DOI:** 10.21956/wellcomeopenres.24838.r96127

**Published:** 2024-10-02

**Authors:** Rayyan Zafar

**Affiliations:** 1 Imperial College London, London, UK

This protocol justifies and sets out the rationale for investigating psilocybin’s effects on Functional Neurological Disorder (FND). The justification is warranted and comes off the back of a mechanistically informed hypothesis linked to proposed amelioration of neurobiological deficits found in FND.

**There are a few minor points/suggestions that could be incorporated which I list below:**

**Additional biomarker integration**

 While the study primarily focuses on neuroimaging and behavioral measures, additional biomarkers may be considered. Current research suggests that biomarkers such as inflammatory markers, or hormonal fluctuations could offer additional insights into how psilocybin affects systemic processes. For example, psychedelics have been shown to modulate immune system activity, including reductions in inflammatory cytokines, which may have relevance to FND’s psychoneuroimmunology model. Including exploratory biomarker analyses could provide a more holistic understanding of psilocybin’s effects. Additionally, inclusion of digital wearables that measure heart rate variability and other autonomic functions may also provide additional insight into the systemic mechanisms of action of psilocybin therapy which may also be core to FND symptomology. Integrating EEG alongside fMRI, could also be done at the same time points and under acute administration to understand effects related to entropy (which was discussed as an exploratory endpoint)

**Gender-specific responses**

 Emerging evidence suggests that gender and hormonal fluctuations may influence responses to psychedelics. This could be particularly important in FND, which disproportionately affects women. Hormonal differences, including those related to menstrual cycles or menopause, could impact serotonergic activity, which plays a crucial role in psilocybin’s effects. The study would benefit from incorporating exploratory analyses of gender-specific responses, potentially tracking hormonal phases in female participants. This could contribute to more personalized treatment approaches for FND.

**Longer follow-up for clinical and fMRI observations**

 The current follow-up period of one week for fMRI scanning may miss long-term functional/structural changes induced by psilocybin. Research indicates that neuroplasticity, particularly in network connectivity, can occur up to 4 weeks post-dosing. Extending the follow-up to 4 weeks or further would allow for a more comprehensive understanding of psilocybin’s longer-term effects on brain connectivity/function in FND. Also extending clinical follow up beyond 3 months is also important to understand the longer-term effects on core clinical symptoms.

 Expanding neuroimaging targets to include networks beyond the DMN, such as the salience network and central executive network, which have been implicated in both FND and psychedelic studies. Also to include DTI analysis as additional to resting state connectivity as Imperial College have data to suggest structural brain changes 4 weeks following a single administration of psilocybin is psychedelic naïve healthy participants in the thalamocortical tracts.

**Psychological support**

 ‘Depending on participant needs we will arrange additional sessions for psychological preparation and integration as required.’ This could induce variability if the primary outcome is powered for imaging, it would be useful to know the parameters under which additional psychological support would be administered. Additionally, there is very limited detail of what will happen in the 3 preparation session and 4 integration session, you mention these are nonspecific to FND but could you clarify in more detail what the aims/objectives of these sessions are. Also, what is the plan for the patients post study, if you are to follow them up would they be receiving treatment as usual?

**Additional scales to consider:**

- Watts connectedness scale
- BEAQ (brief experiential avoidance questionnaire)
- EBI (Emotional breakthrough inventory)
- Psychological Insight scale (PIS) Peill et al
- STAR – Scale to assess therapeutic relationships
- Unpleasant Psychedelic Experience Scale (UPES) score  
- Geneva Emotional Music Scales (GEMS) scores  
- Early Prognostic Predictor Scale (EPPS) total score  
- Imperial psychedelic predictor scale (IPPS)
- Setting questionnaire

Is the study design appropriate for the research question?

Yes

Is the rationale for, and objectives of, the study clearly described?

Yes

Are sufficient details of the methods provided to allow replication by others?

Partly

Are the datasets clearly presented in a useable and accessible format?

Not applicable

Reviewer Expertise:

Neuropsychopharmacology, Psychedelic research, fMRI

I confirm that I have read this submission and believe that I have an appropriate level of expertise to confirm that it is of an acceptable scientific standard.

# Response

**Published:** 2025-03-19

**Authors:** Matt Butler

**Affiliations:** Neuropsychiatry Research and Education Group, King's College London, London, England, UK

Many thanks for your helpful comments which we think have improved the quality of the protocol. We have addressed your suggestions below: 

 1.While the study primarily focuses on neuroimaging and behavioral measures, additional biomarkers may be considered. Current research suggests that biomarkers such as inflammatory markers, or hormonal fluctuations could offer additional insights into how psilocybin affects systemic processes. For example, psychedelics have been shown to modulate immune system activity, including reductions in inflammatory cytokines, which may have relevance to FND’s psychoneuroimmunology model. Including exploratory biomarker analyses could provide a more holistic understanding of psilocybin’s effects. Additionally, inclusion of digital wearables that measure heart rate variability and other autonomic functions may also provide additional insight into the systemic mechanisms of action of psilocybin therapy which may also be core to FND symptomology. Integrating EEG alongside fMRI, could also be done at the same time points and under acute administration to understand effects related to entropy (which was discussed as an exploratory endpoint) Thank you for these suggestions, which are all pertinent to the neurobiology of FND.   Whilst analysis of serum biomarkers would certainly be interesting, we do not feel we have the expertise as a study team to complete these.   We have not included wearable technologies due to previous difficulties we have had in securing institutional approval for their use.   The addition of EEG would be interesting, but perhaps not feasible both in terms of study cost and participant burden.

 2. Emerging evidence suggests that gender and hormonal fluctuations may influence responses to psychedelics. This could be particularly important in FND, which disproportionately affects women. Hormonal differences, including those related to menstrual cycles or menopause, could impact serotonergic activity, which plays a crucial role in psilocybin’s effects. The study would benefit from incorporating exploratory analyses of gender-specific responses, potentially tracking hormonal phases in female participants. This could contribute to more personalized treatment approaches for FND. We agree with the suggestion to conduct exploratory analyses of gender-specific responses.   The sample size is likely too small to render any hormonal or cycle tracking useful.   We do however support the notion in general that gender and hormonal factors are likely to be fruitful avenues of exploration in both FND and psychedelics in future.

 3. The current follow-up period of one week for fMRI scanning may miss long-term functional/structural changes induced by psilocybin. Research indicates that neuroplasticity, particularly in network connectivity, can occur up to 4 weeks post-dosing. Extending the follow-up to 4 weeks or further would allow for a more comprehensive understanding of psilocybin’s longer-term effects on brain connectivity/function in FND. Also extending clinical follow up beyond 3 months is also important to understand the longer-term effects on core clinical symptoms. Thanks for the helpful suggestions.   We considered (and had originally planned) a third fMRI at a later follow-up date, however due to time and financial constraints of this project, we will be unable to do this.   Likewise with clinical follow-ups after 3-months.

 4. Expanding neuroimaging targets to include networks beyond the DMN, such as the salience network and central executive network, which have been implicated in both FND and psychedelic studies. Also to include DTI analysis as additional to resting state connectivity as Imperial College have data to suggest structural brain changes 4 weeks following a single administration of psilocybin is psychedelic naïve healthy participants in the thalamocortical tracts. Based on this suggestion and those from other reviewers, we have expanded our neuroimaging regions of interest beyond the DMN to include e.g. some implicated in the salience network, such as the insulae and anterior cingulate cortices.

 5. ‘Depending on participant needs we will arrange additional sessions for psychological preparation and integration as required.’ This could induce variability if the primary outcome is powered for imaging, it would be useful to know the parameters under which additional psychological support would be administered. Additionally, there is very limited detail of what will happen in the 3 preparation session and 4 integration session, you mention these are nonspecific to FND but could you clarify in more detail what the aims/objectives of these sessions are. Also, what is the plan for the patients post study, if you are to follow them up would they be receiving treatment as usual? Although full discussion of the details of the therapy is beyond the scope of the paper, we have included an additional reference for further reading.   We have included a sentence to qualify that additional sessions would only be used in unforeseen extenuating circumstances.   The plan for patients post-study is mentioned in the ‘End of Study’ section.

 6. Additional scales to consider: Watts connectedness scale BEAQ (brief experiential avoidance questionnaire) EBI (Emotional breakthrough inventory) Psychological Insight scale (PIS) Peill et al STAR – Scale to assess therapeutic relationships Unpleasant Psychedelic Experience Scale (UPES) score  Geneva Emotional Music Scales (GEMS) scores  Early Prognostic Predictor Scale (EPPS) total score  Imperial psychedelic predictor scale (IPPS) Setting questionnaire Thanks for these suggestions. After discussion with our PPI panel, we have now included:   Watts Connectedness Scale Emotional Breakthrough Inventory (also suggested by another reviewer) Psychological Insight Scale Imperial psychedelic predictor scale   We were unable to find the unpleasant psychedelic experiences scale, the early prognosis predictor scale, and the setting questionnaire.   We felt the Geneva Emotional Music Scale went beyond the scope of this protocol.   We have also decided not to include the STAR-C scale.

# Reviewer report: Reviewer response for version 1

**DOI:** 10.21956/wellcomeopenres.24838.r95501

**Published:** 2024-09-22

**Authors:** Lorenzo Pasquini

**Affiliations:** 1 Department of Neurology, University of California, San Francisco, USA

Butler and colleagues propose an open-label clinical in 24 patients with FND to explore potential medium-term mechanisms as well as clinical improvements of 25 mg psilocybin. Overall, the relevance of the problem is clear, and the study design is well described.

**I have following comments which I would like to bring to the attention of the authors:**

1. Altered interoception is  a potential mechanism proposed to be involved in FND and impacted by psilocybin. In this case, the neuroimaging analyses should not only focus on the DMN, but also on the Salience Network, which has been shown to play an important role in interoceptive processes (see Critchley Nat Neurosci 2004 and Seeley JNuerosci 2019).
2. A rationale for using a single dose of 25 mg psilocybin should be provided (vs for example, multiple dosing or lower doses).
3. I would strongly encourage the authors to also acquire multi-shell DTI to map structural connectivity (see also Perez Neuroimage Clinical 2021)
4. The heart beating counting task has several important limitations, notably that participants know they should have about 60 beats/min. A more sophisticated experimental design is needed to accurately measure interoception, where participants are asked to press a button or to blink each time they think they are having a heartbeat. This behavior should be recorded in sync with the EKG. See for example work by Sarah Garfinkel  and Hugo Critchley, as well as by Agustin Ibañes
5. Will the interviews be recorded?
6. A description of the planned statistical analysis is needed (e.g., paired t-test, significance level, correction for multiple comparisons for fMRI, etc.).
7. For fMRI analyses, if interoception is of interest, it may help to add the insula
8. I have a major concern regarding the documentation of adverse events. It is unclear from the study description how adverse events will be collected and how often. For example, it is unclear at which precise time points EKG and pulse will be collected. More details are needed.
9. Another important point is related to screening procedures. Since common symptoms in FND include movement/loss of balance and non-epileptic seizures, it would be important to screen and longitudinally follow participants for fall risk (e.g. using STEDI), as well as ensure through an EEG the absence of epileptic seizures.
10. A final important point relates to the assessment of comorbidities. More details are needed on how patients will be screened for severe depression (threshold used), but also for anxiety and fibromyalgia, to accurately describe the heterogeneity of the disorder
11. Will only psychedelic-naïve participants be recruited? If not, how many previous psychedelics experiences are allowed and how close to screening?
12. The authors should specify whether fMRI analyses will involve global signal regression or not (or both) and describe approaches to mitigate the effects of head movement in the scanner. Levels of sleepiness and anxiety should be assessed after each scan.
13. Will any data be publicly available, e.g. on neuroimaging repositories?

Is the study design appropriate for the research question?

Yes

Is the rationale for, and objectives of, the study clearly described?

Yes

Are sufficient details of the methods provided to allow replication by others?

No

Are the datasets clearly presented in a useable and accessible format?

Partly

Reviewer Expertise:

Neuroimaging, autonomic physiology, psychedelics, interoception, fMRI, neurodegenerative diseases

I confirm that I have read this submission and believe that I have an appropriate level of expertise to confirm that it is of an acceptable scientific standard, however I have significant reservations, as outlined above.

## References

[1] : Neural systems supporting interoceptive awareness. *Nat Neurosci* .2004;7(2) : 10.1038/nn1176 189-95 10.1038/nn1176 14730305

[2] : The Salience Network: A Neural System for Perceiving and Responding to Homeostatic Demands. *J Neurosci* .2019;39(50) : 10.1523/JNEUROSCI.1138-17.2019 9878-9882 10.1523/JNEUROSCI.1138-17.2019 31676604 PMC6978945

[3] : Neuroimaging in Functional Neurological Disorder: State of the Field and Research Agenda. *Neuroimage Clin* .2021;30: 10.1016/j.nicl.2021.102623 102623 10.1016/j.nicl.2021.102623 34215138 PMC8111317

[4] : Knowing your own heart: distinguishing interoceptive accuracy from interoceptive awareness. *Biol Psychol* .2015;104: 10.1016/j.biopsycho.2014.11.004 65-74 10.1016/j.biopsycho.2014.11.004 25451381

[5] : A multidimensional and multi-feature framework for cardiac interoception. *Neuroimage* .2020;212: 10.1016/j.neuroimage.2020.116677 116677 10.1016/j.neuroimage.2020.116677 32101777 PMC7165068

# Response

**Published:** 2025-03-19

**Authors:** Matt Butler

**Affiliations:** Neuropsychiatry Research and Education Group, King's College London, London, England, UK

Many thanks for your helpful comments which we think have improved the quality of the protocol. We have addressed your suggestions below: 

 1.Altered interoception is a potential mechanism proposed to be involved in FND and impacted by psilocybin. In this case, the neuroimaging analyses should not only focus on the DMN, but also on the Salience Network, which has been shown to play an important role in interoceptive processes (see Critchley Nat Neurosci 2004 and Seeley JNuerosci 2019).   Based on this comment, and those of other reviewers, we have now included regions which constitute the salience network, for example the insula and the anterior cingulate cortex.

 2. A rationale for using a single dose of 25 mg psilocybin should be provided (vs for example, multiple dosing or lower doses).   An expanded rationale for choosing 25 mg psilocybin has been included.

 3. I would strongly encourage the authors to also acquire multi-shell DTI to map structural connectivity (see also Perez Neuroimage Clinical 2021)   While we agree that structural connectivity is a potentially sensitive metric, in this particular study we have decided not to include it for the following reasons:   We do not have a non-FND control group to compare to There is little information of short-term psychedelic (or any drug effects) on streamlines Our scan time is limited by feasibility and it will not be possible to add in a full multi-shell DTI sequence for structural connectivity.  

 4. The heart beating counting task has several important limitations, notably that participants know they should have about 60 beats/min. A more sophisticated experimental design is needed to accurately measure interoception, where participants are asked to press a button or to blink each time they think they are having a heartbeat. This behavior should be recorded in sync with the EKG. See for example work by Sarah Garfinkel  and Hugo Critchley, as well as by Agustin Ibañes   We accept that the HTT does have its limitations, some of which have been pointed out here.   Of note, this version of the HTT is a quick and acceptable measure that involves minimal burden on the patient. In the context of this study, this is a benefit, given everything else the participants are being asked to do.   Of note, I don’t think we can take it for granted that most people assume their heartrate is around 60 bpm. There is a likely to be a larger range in both true heartbeat and estimation which ranges from say 50-100bpm.   In this HTT we will explicitly remind participants not to count seconds, and the trials are varied in length. We will also include the TET which will allow us to see how accurate people are at estimating the passage of time.  

 5. Will the interviews be recorded?   We have clarified that the sessions will be audio recorded using Microsoft Teams and stored on a secure cloud server. 3.06 A description of the planned statistical analysis is needed (e.g., paired t-test, significance level, correction for multiple comparisons for fMRI, etc.).   This section has now been updated.

 7. For fMRI analyses, if interoception is of interest, it may help to add the insula   We have now included plans to focus on the insula based on this comment and those from other reviewers.

 8. I have a major concern regarding the documentation of adverse events. It is unclear from the study description how adverse events will be collected and how often. For example, it is unclear at which precise time points EKG and pulse will be collected. More details are needed.   We have added further context to the recording and reporting of adverse events. Recording of all adverse events will take place at every participant contact/visit.   Please note this has been approved by a REC review.   The frequency of e.g. ECG and vital signs are in Fig 2. We have also expanded in the main text which tests are to be undertaken at screening.

 9. Another important point is related to screening procedures. Since common symptoms in FND include movement/loss of balance and non-epileptic seizures, it would be important to screen and longitudinally follow participants for fall risk (e.g. using STEDI), as well as ensure through an EEG the absence of epileptic seizures.   All participants with functional seizures will have their diagnosis confirmed by a neurologist or expert neuropsychiatrist (this is a requirement for the study). Often this will have included a video EEG.   The falls risk point does not to use represent something that needs to be covered specifically for this study, but of course is something clinicians will take into account when screening.

 10. A final important point relates to the assessment of comorbidities. More details are needed on how patients will be screened for severe depression (threshold used), but also for anxiety and fibromyalgia, to accurately describe the heterogeneity of the disorder   Psychiatric disorders will be screened for via The Mini-International Neuropsychiatric Interview.   We will record co-morbid diagnoses at screening.

 11. Will only psychedelic-naïve participants be recruited? If not, how many previous psychedelics experiences are allowed and how close to screening? Please see exclusion criterion 20: “Use of any psychedelic substances >2 times in past 12 months.”   In practice we would not be recruiting participants with frequent or very recent psychedelic use. And our data on self management (Butler et al. 2022)

 12. The authors should specify whether fMRI analyses will involve global signal regression or not (or both) and describe approaches to mitigate the effects of head movement in the scanner. Levels of sleepiness and anxiety should be assessed after each scan.   The primary analysis will not include global signal regression because of the issues raised and its covariance.

 13. Will any data be publicly available, e.g. on neuroimaging repositories? We have now stated that all shareable study data, including sharable neuroimaging data, will be uploaded to an online sharing platform hosted by King’s College London.

# Reviewer report: Reviewer response for version 1

**DOI:** 10.21956/wellcomeopenres.24838.r96124

**Published:** 2024-09-17

**Authors:** Manoj Doss

**Affiliations:** 1 Department of Psychiatry and Behavioral Sciences, The University of Texas at Austin Dell Medical School, Austin, USA

Butler et al. describe the procedures for a future study on the effects of psilocybin on brain function (as measured with fMRI), as well as clinical outcomes, in functional neurological disorder (FND). The overall design and methods are fairly straightforward. My main concern is that some of the background literature doesn’t really support the hypotheses proposed here, which seem to be more specific to narratives in psychedelic research rather than a bridge between idiosyncratic psychedelic effects and abnormalities in FND.

**Below are my specific points:**

 1) Fourth paragraph of the Introduction, the predictive processing section is rather non-specific. In general, the predictive processing framework has become a bit of a catch-all for every disorder and every drug effect.

 2) First paragraph of Mechanistic evidence in FND, “ *People with FND have shown increased connectivity in brain regions which comprise the default mode network (DMN), such as the medial prefrontal cortex and posterior cingulate cortex (PCC), both in resting-state and task based paradigms ^19–25^*.” I only skimmed these references, but I think this claim is largely unsupported. Reference 19 didn’t find increased FC within the DMN but rather increased FC between motor cortex and DMN regions. Reference 20 didn’t look at FC. Reference 21 didn’t find increased FC among DMN regions. Reference 22 also didn’t look at FC. Reference 23 yet again didn’t look at FC. Reference 24, which was a SPECT study (and thus difficult to look at FC), didn’t look at FC, and they found *less* activity in the DMN. Reference 25 didn’t look at FC. Given that much of this proposal relies on the DMN, the lack of DMN evidence is rather problematic. Some (but not all) of the studies above found changes in vmPFC activation (though they might not all have been directionally consistent), so it might be easier to focus on vmPFC or other specific brain areas (that may not necessarily be part of the DMN).

 3) Third paragraph of Current psychedelic research, “ *Modern psychedelic research involves non-directive psychological support ^39,40,56,57^, in which the therapeutic relationship between participant and therapist is of central importance ^58^.*” The importance of the patient-therapist relationship to psychedelic therapy is currently conjecture, and reference 58 is not exactly the most evidence-based text. Likewise, “ *At doses of 25mg psilocybin, participants may have transient psychologically-challenging experiences ^59^, however psychological preparation can mitigate these…*” Again, I’m not sure there’s good evidence yet for the degree to which preparation can influence challenging experiences. I think more nuanced language is needed for these assumptions that are at times taken as fact in psychedelic research.

 4) First paragraph of Mechanistic effects of psychedelics, “ *The extent of reduction in connectivity correlates with self-reported degree of loss of selfhood (‘ego-dissolution’) ^63^*.” This effect has yet to replicate, and in a study with a larger *N* and less motion artifacts, the correlation between attenuated DMN FC and ego dissolution was basically non-existent at r = .06 (https://doi.org/10.1016/j.nicl.2018.03.005) ^1^ . Moreover, “ *Functional neuroimaging in psychedelics have shown relatively consistent effects of decreases in within-network-connectivity and increases in between-network-connectivity in canonical brain networks including the DMN ^64^.*” These findings are not really that consistent when one considers the specific networks impacted (again see https://doi.org/10.1016/j.nicl.2018.03.005 ^1^ for the lack of agreement between which 2 specific networks increased their FC across 3 studies).

 5) Second paragraph of Mechanistic effects of psychedelics, “ *Loosened global network connectivity may enable improvement of neuropsychiatric symptoms by reducing the influence of top-down processing and loosening established prediction errors ^66^.*” I’m not sure what is meant by “ *loosening established prediction errors*.” Moreover, while within-network FC under psychedelics is reduced (under resting-state conditions), there are increases in between-network FC with at least one study finding increases between the DMN and SM networks (https://doi.org/10.1073/pnas.2218949120) ^2^ . Given that reference 19 mentions that there are increases in FC between motor cortex and the DMN in FND, I’m not sure how to reconcile these two sets of findings.

 Another issue is that these are acute effects. If one turns to the literature on persisting resting-state effects of psychedelics on FC, then it is unclear what the implications are for FND, as the effects are rather unreliable such as increases in DMN FC (https://doi.org/10.1038/s41598-017-13282-7) ^3^ , decreases in DMN FC (https://doi.org/10.1177/0269881120909409 ^4^ , https://doi.org/10.1016/j.neuroimage.2019.04.009 ^5^ ), no impact on DMN FC (https://doi.org/10.1038/s41398-021-01706-y) ^6^ , and even both increase and decreases in DMN FC dependent on the analysis (https://doi.org/10.1038/s41591-022-01744-z) ^7^ , though perhaps these inconsistencies in DMN FC have little bearing on FND considering the above cited studies in which DMN FC is rarely impacted in FND.

 6) If the researchers’ interest is in the DMN, why not probe function known to be supported by the DMN such as episodic memory, semantic memory, future simulation, theory of mind, or even attentional tasks with inappropriate DMN engagement that is thought to support mind wandering?

 7) Regarding the endpoints, if one’s interest is in intrinsic FC (i.e., what resting-state neuroimaging aims to capture), then resting-state analyses of the task-based data should produce similar results. That is, if one is attempting to capture a general scaffolding of functional brain activity, then it should be largely insensitive to task manipulations, and if these effects are abolished by some stimuli and button presses, then what was being measured wasn’t intrinsic in the first place. It’s possible that the variability in cognitive operations of the resting-state is a likely culprit for the unreliable acute and post-acute effects of psychedelics, as well as the unreliability in various disorders (e.g., large studies finding both increases and decreases in DMN FC in depression, https://doi.org/10.1001/jamapsychiatry.2015.0071 ^8^ , https://doi.org/10.1073/pnas.1900390116 ^9^ ). Finding similar post-acute effects of psychedelics on FC under diverse cognitive constraints would provide stronger support that these FC changes are reliable markers. Analyzing task-based data with resting-state analyses is something that has been done (e.g., https://doi.org/10.1073/pnas.1608819113) ^10^ , can improve predictive models (e.g., https://doi.org/10.1038/nn.4179) ^11^ , and has been advocated for (e.g., https://doi.org/10.1016/j.tics.2021.09.005) ^12^ .

 8) First bullet point of Hypotheses under investigation, “ *The direction of change will be towards that of healthy controls (Figure 1).*” Without a matched control group, it’ll be hard to determine this. Moreover, I’m not sure how Figure 1 is related to this point.

 9) It’s a bit odd that this manuscript discusses how DMN FC is increased in FND, yet the effect that is used to justify the sample size is an increase in FC following psilocybin therapy (i.e., reference ^76^).

 10) Resting-state scans with eyes closed can be problematic, as participants might fall asleep. In a fairly large study with eyes open conditions, >40% of participants reported falling asleep (https://doi.org/10.1016/j.jad.2019.07.066) ^13^ , a number that could be potentially larger when one considers that people do not always realize that they fall asleep, and some participants may have not wanted to upset researchers. Thus, with eye-closed conditions, it’s not impossible that up to 50% of participants could fall asleep. I’d recommend a fixation cross instead or even the Inscapes video, as it better standardizes the perceptual input (though not necessarily the cognitive operations) across participants.

 11) There doesn’t seem to be much information on the plan for analyzing the task-based data.

 12) Regarding the follow-up, it might be worth examining a later time-point (e.g., 1 year) for clinical assessments, as one issue raised in regarding the recent rejection of MDMA therapy was that the trials didn’t have longer term follow-ups.

Is the study design appropriate for the research question?

Partly

Is the rationale for, and objectives of, the study clearly described?

Partly

Are sufficient details of the methods provided to allow replication by others?

Partly

Are the datasets clearly presented in a useable and accessible format?

Not applicable

Reviewer Expertise:

Psychedelics

I confirm that I have read this submission and believe that I have an appropriate level of expertise to confirm that it is of an acceptable scientific standard, however I have significant reservations, as outlined above.

## References

[1] : Altered network hub connectivity after acute LSD administration. *Neuroimage Clin* .2018;18: 10.1016/j.nicl.2018.03.005 694-701 10.1016/j.nicl.2018.03.005 29560311 PMC5857492

[2] : Human brain effects of DMT assessed via EEG-fMRI. *Proc Natl Acad Sci U S A* .2023;120(13) : 10.1073/pnas.2218949120 e2218949120 10.1073/pnas.2218949120 36940333 PMC10068756

[3] : Psilocybin for treatment-resistant depression: fMRI-measured brain mechanisms. *Sci Rep* .2017;7(1) : 10.1038/s41598-017-13282-7 13187 10.1038/s41598-017-13282-7 29030624 PMC5640601

[4] : Subacute effects of the psychedelic ayahuasca on the salience and default mode networks. *J Psychopharmacol* .2020;34(6) : 10.1177/0269881120909409 623-635 10.1177/0269881120909409 32255395

[5] : Psilocybin-assisted mindfulness training modulates self-consciousness and brain default mode network connectivity with lasting effects. *Neuroimage* .2019;196: 10.1016/j.neuroimage.2019.04.009 207-215 10.1016/j.neuroimage.2019.04.009 30965131

[6] : Psilocybin therapy increases cognitive and neural flexibility in patients with major depressive disorder. *Transl Psychiatry* .2021;11(1) : 10.1038/s41398-021-01706-y 574 10.1038/s41398-021-01706-y 34750350 PMC8575795

[7] : Increased global integration in the brain after psilocybin therapy for depression. *Nat Med* .2022;28(4) : 10.1038/s41591-022-01744-z 844-851 10.1038/s41591-022-01744-z 35411074

[8] : Large-Scale Network Dysfunction in Major Depressive Disorder: A Meta-analysis of Resting-State Functional Connectivity. *JAMA Psychiatry* .2015;72(6) : 10.1001/jamapsychiatry.2015.0071 603-11 10.1001/jamapsychiatry.2015.0071 25785575 PMC4456260

[9] : Reduced default mode network functional connectivity in patients with recurrent major depressive disorder. *Proc Natl Acad Sci U S A* .2019;116(18) : 10.1073/pnas.1900390116 9078-9083 10.1073/pnas.1900390116 30979801 PMC6500168

[10] : Dynamic brain network reconfiguration as a potential schizophrenia genetic risk mechanism modulated by NMDA receptor function. *Proc Natl Acad Sci U S A* .2016;113(44) : 10.1073/pnas.1608819113 12568-12573 10.1073/pnas.1608819113 27791105 PMC5098640

[11] : A neuromarker of sustained attention from whole-brain functional connectivity. *Nat Neurosci* .2016;19(1) : 10.1038/nn.4179 165-71 10.1038/nn.4179 26595653 PMC4696892

[12] : Is it time to put rest to rest?. *Trends Cogn Sci* .2021;25(12) : 10.1016/j.tics.2021.09.005 1021-1032 10.1016/j.tics.2021.09.005 34625348 PMC8585722

[13] : Unstable wakefulness during resting-state fMRI and its associations with network connectivity and affective psychopathology in young adults. *J Affect Disord* .2019;258: 10.1016/j.jad.2019.07.066 125-132 10.1016/j.jad.2019.07.066 31401540 PMC6710159

# Response

**Published:** 2025-03-19

**Authors:** Matt Butler

**Affiliations:** Neuropsychiatry Research and Education Group, King's College London, London, England, UK

Many thanks for your helpful comments which we think have improved the quality of the protocol.

 We have addressed your suggestions below: 

**Comment: **2.01 Fourth paragraph of the Introduction, the predictive processing section is rather non-specific. In general, the predictive processing framework has become a bit of a catch-all for every disorder and every drug effect.

**Response: **Thank you. Based on this comment and those from other reviewers we have removed mention of the predictive processing framework.

**Comment:** 2.02 First paragraph of Mechanistic evidence in FND, “People with FND have shown increased connectivity in brain regions which comprise the default mode network (DMN), such as the medial prefrontal cortex and posterior cingulate cortex (PCC), both in resting-state and task based paradigms19–25.” I only skimmed these references, but I think this claim is largely unsupported. Reference 19 didn’t find increased FC within the DMN but rather increased FC between motor cortex and DMN regions. Reference 20 didn’t look at FC. Reference 21 didn’t find increased FC among DMN regions. Reference 22 also didn’t look at FC. Reference 23 yet again didn’t look at FC. Reference 24, which was a SPECT study (and thus difficult to look at FC), didn’t look at FC, and they found less activity in the DMN. Reference 25 didn’t look at FC. Given that much of this proposal relies on the DMN, the lack of DMN evidence is rather problematic. Some (but not all) of the studies above found changes in vmPFC activation (though they might not all have been directionally consistent), so it might be easier to focus on vmPFC or other specific brain areas (that may not necessarily be part of the DMN).

**Response:** Based on this comment and those from other reviewers we have removed explicit mention of the DMN and are instead focussing on specific regions from the FND and psychedelic literature (such as the mPFC).

**Comment: **2.03 Third paragraph of Current psychedelic research, “Modern psychedelic research involves non-directive psychological support39,40,56,57, in which the therapeutic relationship between participant and therapist is of central importance58.” The importance of the patient-therapist relationship to psychedelic therapy is currently conjecture, and reference 58 is not exactly the most evidence-based text. Likewise, “At doses of 25mg psilocybin, participants may have transient psychologically-challenging experiences59, however psychological preparation can mitigate these…” Again, I’m not sure there’s good evidence yet for the degree to which preparation can influence challenging experiences. I think more nuanced language is needed for these assumptions that are at times taken as fact in psychedelic research.

**Response:** Thanks for bringing up this discussion, which is an active topic in the psychedelic research literature at the moment.   Although we agree conclusive evidence for the psychological component is currently not available, it is, in our opinion, the safest course of action for a study such as this. We’d welcome further exploration in other studies.  We have toned down the language as suggested.

**Comment:** 2.04 First paragraph of Mechanistic effects of psychedelics, “The extent of reduction in connectivity correlates with self-reported degree of loss of selfhood (‘ego-dissolution’)63.” This effect has yet to replicate, and in a study with a larger N and less motion artifacts, the correlation between attenuated DMN FC and ego dissolution was basically non-existent at r = .06 (https://doi.org/10.1016/j.nicl.2018.03.005)1. Moreover, “Functional neuroimaging in psychedelics have shown relatively consistent effects of decreases in within-network-connectivity and increases in between-network-connectivity in canonical brain networks including the DMN64.” These findings are not really that consistent when one considers the specific networks impacted (again see https://doi.org/10.1016/j.nicl.2018.03.0051 for the lack of agreement between which 2 specific networks increased their FC across 3 studies).

**Response: **We have now removed the sentence on correlation with ego dissolution and have toned down the language on the DMN.    

**Comment: **2.05 Second paragraph of Mechanistic effects of psychedelics, “Loosened global network connectivity may enable improvement of neuropsychiatric symptoms by reducing the influence of top-down processing and loosening established prediction errors66.” I’m not sure what is meant by “loosening established prediction errors.” Moreover, while within-network FC under psychedelics is reduced (under resting-state conditions), there are increases in between-network FC with at least one study finding increases between the DMN and SM networks (https://doi.org/10.1073/pnas.2218949120)2. Given that reference 19 mentions that there are increases in FC between motor cortex and the DMN in FND, I’m not sure how to reconcile these two sets of findings.

**Response:** Given we have now removed mention of the predictive processing theory of FND, we have also removed the parts quoted in this suggestion.    

**Comment: **2.06 Another issue is that these are acute effects. If one turns to the literature on persisting resting-state effects of psychedelics on FC, then it is unclear what the implications are for FND, as the effects are rather unreliable such as increases in DMN FC (https://doi.org/10.1038/s41598-017-13282-7)3, decreases in DMN FC (https://doi.org/10.1177/02698811209094094, https://doi.org/10.1016/j.neuroimage.2019.04.0095), no impact on DMN FC (https://doi.org/10.1038/s41398-021-01706-y)6, and even both increase and decreases in DMN FC dependent on the analysis (https://doi.org/10.1038/s41591-022-01744-z)7, though perhaps these inconsistencies in DMN FC have little bearing on FND considering the above cited studies in which DMN FC is rarely impacted in FND.  

**Response: **We agree there is a lot of inconsistency in these findings and this is part of our reasoning to move away from limiting our hypotheses to the DMN.  As a caveat, however, these studies generally pertain to the acute effects of psychedelics, something that we are not looking at in this study.

**Comment:** 2.07 If the researchers’ interest is in the DMN, why not probe function known to be supported by the DMN such as episodic memory, semantic memory, future simulation, theory of mind, or even attentional tasks with inappropriate DMN engagement that is thought to support mind wandering?

**Response:** Thanks for these suggestions. We have now toned down our emphasis on probing the DMN. Many of these functions would only be indirectly associated with FND (except perhaps for measures of attention).

**Comment:** 2.08 Regarding the endpoints, if one’s interest is in intrinsic FC (i.e., what resting-state neuroimaging aims to capture), then resting-state analyses of the task-based data should produce similar results. That is, if one is attempting to capture a general scaffolding of functional brain activity, then it should be largely insensitive to task manipulations, and if these effects are abolished by some stimuli and button presses, then what was being measured wasn’t intrinsic in the first place. It’s possible that the variability in cognitive operations of the resting-state is a likely culprit for the unreliable acute and post-acute effects of psychedelics, as well as the unreliability in various disorders (e.g., large studies finding both increases and decreases in DMN FC in depression, https://doi.org/10.1001/jamapsychiatry.2015.00718, https://doi.org/10.1073/pnas.19003901169). Finding similar post-acute effects of psychedelics on FC under diverse cognitive constraints would provide stronger support that these FC changes are reliable markers. Analyzing task-based data with resting-state analyses is something that has been done (e.g., https://doi.org/10.1073/pnas.1608819113)10, can improve predictive models (e.g., https://doi.org/10.1038/nn.4179)11, and has been advocated for (e.g., https://doi.org/10.1016/j.tics.2021.09.005)12.

**Response: **While we take into account the comments on the connectivity patterns across a task, which may yield similar results as the resting state, we would see this as a post-hoc analysis of the task fMRI data.   To this end, we will conduct an exploratory replication analysis of the task data, but analysed in the same manner as the resting state (including task regressors).

 Comment: 2.09 First bullet point of Hypotheses under investigation, “The direction of change will be towards that of healthy controls (Figure 1).” Without a matched control group, it’ll be hard to determine this. Moreover, I’m not sure how Figure 1 is related to this point.

**Response:** Apologies, we mentioned fig 1 in error. Reference has been removed from the sentence.

**Comment:** 2.10 It’s a bit odd that this manuscript discusses how DMN FC is increased in FND, yet the effect that is used to justify the sample size is an increase in FC following psilocybin therapy (i.e., reference 76).

**Response: **Thanks for this point which further illustrates the complexities of the literature.   The narrative we had been building was around acute effects on DMN connectivity (not longer-term effects, as shown in this paper).   Nevertheless, this narrative has now been removed,  We have stuck with this measure as an (imperfect) means of calculating sample size given our ongoing desire to assess network connectivity of the mPFC.

 2.11 Resting-state scans with eyes closed can be problematic, as participants might fall asleep. In a fairly large study with eyes open conditions, >40% of participants reported falling asleep (https://doi.org/10.1016/j.jad.2019.07.066)13, a number that could be potentially larger when one considers that people do not always realize that they fall asleep, and some participants may have not wanted to upset researchers. Thus, with eye-closed conditions, it’s not impossible that up to 50% of participants could fall asleep. I’d recommend a fixation cross instead or even the Inscapes video, as it better standardizes the perceptual input (though not necessarily the cognitive operations) across participants.

**Response: **We have updated the methods to specify that we will be using a fixation cross or the animation video during the rs-fMRI.

**Comment:** 2.12 There doesn’t seem to be much information on the plan for analyzing the task-based data.

**Response: **This section has been updated to include further information on analysing the task-based data (both the timings and the fMRI results).

**Comment: **2.13 Regarding the follow-up, it might be worth examining a later time-point (e.g., 1 year) for clinical assessments, as one issue raised in regarding the recent rejection of MDMA therapy was that the trials didn’t have longer term follow-ups.

**Response: **Thanks for this suggestion, which has also been raised by other reviewers. Given the time and financial constraints of this study, a 1-year follow-up unfortunately won’t be feasible. As this study is not a clinical trial, the results cannot form part of any licencing decisions.

# Reviewer report: Reviewer response for version 1

**DOI:** 10.21956/wellcomeopenres.24838.r93854

**Published:** 2024-09-12

**Authors:** Manesh Girn

**Affiliations:** 1 Department of Neurology, University of California San Francisco, San Francisco, CA, USA

The present manuscript proposes a study protocol for a mechanistic study that applies functional magnetic resonance imaging (fMRI) and a battery of psychometric assessments to assess the mechanisms by which psilocybin might help treat functional neurological disorders (FND). It aims to both help illuminate the mechanisms underlying FND and its potential alleviation, while simultaneously assessing whether psychedelic administration is safe and well-tolerated by this patient population. Resting-state fMRI analyses are centered primarily on regions of interest that overlap with the default mode network, and task-based fMRI analyses are aimed at assessing changes in the neural underpinnings of motor agency.

 Overall, this an important preliminary study on a novel indication for psilocybin therapy and has strong potential to yield valuable mechanistic and clinical insights. That said, there are a few things the authors might want to consider in terms of their specific analyses and hypotheses.

- The authors state that “a representative sample of motor and seizure subtypes will be sought for inclusion”. However, the majority of the narrative, cited research, and focus seems to be biased towards functional motor disorders. Indeed, there is no explicit mention of functional seizures throughout. Moreover, there is no explicit mention of existing overarching theoretical models for the neurobiology of either.
  - Do the authors assume that the pathophysiology is the same for both of these conditions?
  - Might it be better to have distinct theoretically and empirically motivated hypotheses for each?
  - For example, in our recent paper ( https://doi.org/10.1177/02698811241248395) Lewis *et al*.(2024 ^1^ ) we outline hypotheses and fMRI empirical directions for functional seizures in particular based on past theoretical models and findings (e.g., the Integrated Cognitive Model).
- Relatedly, the authors describe predictive processing but don’t clearly offer an explanation as to why the intended analyses/hypotheses are consistent with or might shed light on this perspective on FND.
- Moreover, the mechanistic hypotheses as currently articulated are relatively non-specific and not clearly motivated. More specific hypotheses and explicit rationales for the regions of interest chosen for the seed-based analyses, and for the analyses conduced for the motor agency task, would be ideal.
  - Greater explicit reference to previous theoretical models (of FND mechanisms and/or psilocybin therapeutic mechanisms) to support the hypotheses/ROI selection would also be a strength.
- The authors could have also provided much more detail on the specific statistical/analytical approaches that will be conducted for the seed-based analyses (e.g., as pertains to cluster correction/multiple comparison correction, how the coordinates of ROIs will be determined, etc.).
- Furthermore, the papers cited in support of the idea that the DMN is of central importance for FND (15, 19-25) do not clearly support focusing on this network over others. Several papers highlight the ventromedial prefrontal cortex in particular, but I do not see compelling evidence that the DMN per se is critical. A large literature highlights the VMPFC’s role in action selection and decision making – might that role be of particular importance, as opposed to its membership in the DMN per se?
  - It also should be emphasized that the link between the DMN and psychedelic ego dissolution is far from unequivocal, and psychedelic mechanisms cannot be primarily attributed to the DMN based on existing empirical evidence, strictly speaking. A more distributed, dynamic, multi-network explanation is likely more apt (e.g., as we have argued in Girn *et al. *(2023 ^2^ ), *Trends in Cognitive Science*).
  - In addition, given the emphasis on interoceptive awareness, somatic signalling, and agency/body ownership, the insula presents itself as an essential region of interest for seed-based analyses.
  - Motor cortex functional connectivity would also presumably be of particular interest, as it has been previously implicated in FND (e.g., *J Neurology Neurosurgery Psychiatry, *Diez *et al. (*2019 ^3^ ); *Molecular Psychiatry, *Diez *et al*. (2021 ^4^ )).
- Although perhaps more advanced and exploratory, the authors might consider dynamic functional connectivity analyses, such as those which seek to estimate brain states and their dynamic evolution to characterize neural changes related to FND (e.g., see *Network Neuroscience, *Lurie *et al.* (2020 ^5^ )).
  - Indeed, FND is a dynamic condition in which symptoms seem to occur in a quasi-periodic fashion (e.g., seizure episodes or dystonic movements), and which therefore might exhibit pathophysiology particularly in the way in which the brain traverses dynamic interactions between networks (i.e., in the dynamic changes within a scan rather than the average across the scan as a whole).
    - E.g., there may be less time spent in patterns exhibiting strong connectivity between the VMPFC, motor cortex, and/or the insula for those with a functional movement disorder - and/or there might be cyclic/recurring transient patterns of motor cortex (dis)connectivity in such patients that would not be detectable with time-averaged analyses.
    - We also touch on the importance of dynamic, systems-level analyses in our functional seizures paper linked above.
- The authors selected a strong comprehensive suite of non-neural measures to assess relevant psychological and clinical constructs at pre, immediately post-acute, and follow-up timepoints.
  - One suggestion for measures of the acute experience is to consider using the Emotional Breakthrough Inventory, (Roseman *et al*. 2019 ^6^ ), *Journal of Psychopharmacology*) especially given the potential role of emotional abreaction in such conditions.
  - The authors might also consider a simple measure of seizure occurrences/frequency in the case of functional seizure patients.

All in all, with more clearly and cogently motivated hypotheses and analytic approaches, this study has potential to provide an important contribution that expands the breadth of psilocybin therapy, and which may also shed valuable light on the mechanisms of FND and its treatment.

Is the study design appropriate for the research question?

Yes

Is the rationale for, and objectives of, the study clearly described?

Yes

Are sufficient details of the methods provided to allow replication by others?

Partly

Are the datasets clearly presented in a useable and accessible format?

Yes

Reviewer Expertise:

functional neuroimaging/fMRI, large-scale brain networks, static and dynamic functional connectivity analyses, psychedelic neuroimaging, default mode network

I confirm that I have read this submission and believe that I have an appropriate level of expertise to confirm that it is of an acceptable scientific standard, however I have significant reservations, as outlined above.

## References

[1] : Exploring psychedelic-assisted therapy in the treatment of functional seizures: A review of underlying mechanisms and associated brain networks. *J Psychopharmacol* .2024;38(5) : 10.1177/02698811241248395 407-416 10.1177/02698811241248395 38654554 PMC11102649

[2] : A complex systems perspective on psychedelic brain action. *Trends Cogn Sci* .2023;27(5) : 10.1016/j.tics.2023.01.003 433-445 10.1016/j.tics.2023.01.003 36740518

[3] : Corticolimbic fast-tracking: enhanced multimodal integration in functional neurological disorder. *J Neurol Neurosurg Psychiatry* .2019;90(8) : 10.1136/jnnp-2018-319657 929-938 10.1136/jnnp-2018-319657 30850473 PMC6625895

[4] : Early-life trauma endophenotypes and brain circuit-gene expression relationships in functional neurological (conversion) disorder. *Mol Psychiatry* .2021;26(8) : 10.1038/s41380-020-0665-0 3817-3828 10.1038/s41380-020-0665-0 32051548 PMC7423688

[5] : Questions and controversies in the study of time-varying functional connectivity in resting fMRI. *Netw Neurosci* .2020;4(1) : 10.1162/netn_a_00116 30-69 10.1162/netn_a_00116 32043043 PMC7006871

[6] : Emotional breakthrough and psychedelics: Validation of the Emotional Breakthrough Inventory. *J Psychopharmacol* .2019;33(9) : 10.1177/0269881119855974 1076-1087 10.1177/0269881119855974 31294673

# Response

**Published:** 2025-03-19

**Authors:** Matt Butler

**Affiliations:** Neuropsychiatry Research and Education Group, King's College London, London, England, UK

Many thanks for your helpful comments which we think have improved the quality of the protocol. We have addressed your suggestions below:

**Comment: **1.01 The authors state that “a representative sample of motor and seizure subtypes will be sought for inclusion”.

**Response: **We have specified that this will be a roughly 50/50 split between functional seizures and functional motor disorder, with allowed variance to account for the small sample size.

**Comment:** 1.02 The majority of the narrative, cited research, and focus seems to be biased towards functional motor disorders. Indeed, there is no explicit mention of functional seizures throughout. Moreover, there is no explicit mention of existing overarching theoretical models for the neurobiology of either.   Do the authors assume that the pathophysiology is the same for both of these conditions?   Might it be better to have distinct theoretically and empirically motivated hypotheses for each? For example, in our recent paper (https://doi.org/10.1177/02698811241248395) Lewis et al.(20241) we outline hypotheses and fMRI empirical directions for functional seizures in particular based on past theoretical models and findings (e.g., the Integrated Cognitive Model).

**Response:** We have suggested that whilst there may well be distinct underlying pathophysiology between FND subtypes (particularly between motor and seizure subtypes), that their common co-occurrence suggests that there may also be overlap.   Although future research can and should look at mechanisms underlying subtypes in more granular detail, this is not possible within the scope of this study.   We have made reference to the integrated cognitive model in our discussion and have cited the helpfully suggested relevant paper.

**Comment:** 1.03 Relatedly, the authors describe predictive processing but don’t clearly offer an explanation as to why the intended analyses/hypotheses are consistent with or might shed light on this perspective on FND.

**Response:** This section has been removed to improve clarity.

**Comment: **1.04 Moreover, the mechanistic hypotheses as currently articulated are relatively non-specific and not clearly motivated. More specific hypotheses and explicit rationales for the regions of interest chosen for the seed-based analyses, and for the analyses conduced for the motor agency task, would be ideal.   Greater explicit reference to previous theoretical models (of FND mechanisms and/or psilocybin therapeutic mechanisms) to support the hypotheses/ROI selection would also be a strength.

**Response:** We have now updated our ROI specification to bring it up to date with the current FND functional neuroimaging literature.   A theoretical justification for each region is probably beyond the scope of this protocol paper, particularly as the analyses are exploratory.

**Comment:** 1.05 The authors could have also provided much more detail on the specific statistical/analytical approaches that will be conducted for the seed-based analyses (e.g., as pertains to cluster correction/multiple comparison correction, how the coordinates of ROIs will be determined, etc.).

**Response:** We have updated the analysis methods to explain that for each seed we will conduct a seed to voxel analysis using permutation-based multiple comparisons correction at the cluster level as implemented within the CONN toolbox with white matter and CSF (but not grey matter) regressors.

**Comment:** 1.06 Furthermore, the papers cited in support of the idea that the DMN is of central importance for FND (15, 19-25) do not clearly support focusing on this network over others. Several papers highlight the ventromedial prefrontal cortex in particular, but I do not see compelling evidence that the DMN per se is critical. A large literature highlights the VMPFC’s role in action selection and decision making – might that role be of particular importance, as opposed to its membership in the DMN per se?

**Response:** Based on this suggestion and from those made by other reviewers, we have decided to move focus away from the default mode network in general, and move instead towards the specific regions in particular.

**Comment: **1.07 It also should be emphasized that the link between the DMN and psychedelic ego dissolution is far from unequivocal, and psychedelic mechanisms cannot be primarily attributed to the DMN based on existing empirical evidence, strictly speaking. A more distributed, dynamic, multi-network explanation is likely more apt (e.g., as we have argued in Girn et al. (20232), Trends in Cognitive Science).

**Response: **We agree with this suggestion that it is more complicated than simply the DMN. We have updated the introduction to reflect this and have included the helpful reference.

**Comment:** 1.08 In addition, given the emphasis on interoceptive awareness, somatic signalling, and agency/body ownership, the insula presents itself as an essential region of interest for seed-based analyses.

**Response:** Motor cortex functional connectivity would also presumably be of particular interest, as it has been previously implicated in FND (e.g., J Neurology Neurosurgery Psychiatry, Diez et al. (20193); Molecular Psychiatry, Diez et al. (20214)). We agree and have added these regions to the analysis plan.

**Comment: **1.09 Although perhaps more advanced and exploratory, the authors might consider dynamic functional connectivity analyses, such as those which seek to estimate brain states and their dynamic evolution to characterize neural changes related to FND (e.g., see Network Neuroscience, Lurie et al. (20205)).  

**Response: **We agree with the reviewer that dynamic connectivity would be an interesting analysis and the data will be suitable for this, but the study plan does not currently include any hypotheses for such an analysis and after reflecting on this we are not sure what they would be.   We could certainly derive some from the psychedelic literature, but the link to FND is exploratory. Therefore, we have decided not to include this as part of our protocol but recognise the value of exploratory analyses. We also note our data will be shared.

**Comment:** 1.10 One suggestion for measures of the acute experience is to consider using the Emotional Breakthrough Inventory, (Roseman et al. 20196), Journal of Psychopharmacology) especially given the potential role of emotional abreaction in such conditions.

**Response:** This has now been included.

**Comment:** 1.11 The authors might also consider a simple measure of seizure occurrences/frequency in the case of functional seizure patients.

**Response: **We have clarified that we will be recording participant reports of average number of seizures in intervening period when undertaking the CGI. Likewise, simple descriptions of motor/other symptoms will be recorded. Any further formal recording will be beyond the scope of this study, which is not a clinical trial.
